# Afraid and tired: A longitudinal study of the relationship between cancer‐related fatigue and fear of cancer recurrence in long‐term cancer survivors

**DOI:** 10.1002/cam4.7313

**Published:** 2024-06-07

**Authors:** Geneviève Trudel, Sophie Lebel, Robert L. Stephens, Caroline Séguin Leclair, Corinne R. Leach, J. Lee Westmaas

**Affiliations:** ^1^ School of Psychology University of Ottawa Ottawa Ontario Canada; ^2^ ICF International Inc Atlanta Georgia USA; ^3^ Moffit Cancer Center Tampa Florida USA; ^4^ American Cancer Society Atlanta Georgia USA

**Keywords:** age, cancer survivors, cancer‐related fatigue, fear of cancer recurrence, longitudinal

## Abstract

**Objective:**

Cancer‐related fatigue (CRF) and fear of cancer recurrence (FCR) are two common concerns experienced by cancer survivors. However, the relationship between these two concerns is poorly understood, and whether CRF and FCR influence each other over time is unclear.

**Methods:**

Data were from a national, prospective, longitudinal study, the American Cancer Society's Study of Cancer Survivors‐I (SCS‐I). Surveys were completed by 1395 survivors of 10 different cancer types at three time‐points, including assessment 1.3 years (T1), 2.2 years (T2) and 8.8 years (T3) following their cancer diagnosis. CRF was assessed using the fatigue‐inertia subscale of the Profile of Mood States, and FCR by the FCR subscale of the Cancer Problems in Living Scale. Multiple group random intercepts cross‐lagged panel models investigated prospective associations between CRF and FCR.

**Results:**

For younger participants (at or below median age of 55 years, *n* = 697), CRF at T1 and T2 marginally and significantly predicted FCR at T2 and T3, respectively, but no lagged effects of FCR on subsequent CRF were observed. Cross‐lagged effects were not observed for survivors over 55 years of age.

**Conclusion:**

Both CRF and FCR are debilitating side effects of cancer and its treatments. Given that CRF may be predictive of FCR, it possible that early detection and intervention for CRF could contribute to lowering FCR severity.

## 1 INTRODUCTION

By 2030 the prevalence of cancer diagnoses in Canada is expected to increase by 80% compared to 2005 levels,^1^ and by 131% in the United States. compared to levels from 2019.^2^ Fortunately, screening, early detection, and increased treatment options have resulted in decreased cancer mortality rates.^3^ Today, the predicted 5‐year survival rate is 64%.^1^ However, a longer survival period often entails strenuous treatment regimens and debilitating symptoms such as fatigue.^4^

Cancer‐related fatigue (CRF) is a multidimensional phenomenon that can have physical, mental, and emotional manifestation.^4^, ^5^ CRF is defined as “a distressing, persistent, subjective sense of physical, emotional, and/or cognitive tiredness or exhaustion related to cancer or cancer treatment that is not proportional to recent activity and interferes with usual functioning.”^6^ Around 52% of cancer patients report having experienced CRF over the course of their diagnosis,^7^ and its presence can result in a significant decline in patients' quality of life.^8^ CRF's etiology, however, is currently not well understood, with possible causes including cancer treatments, cancer itself, genetic predispositions, and/or environmental factors.^5^, ^9^ Unfortunately, CRF is one of the many symptoms that commonly persists beyond cancer diagnosis and treatment. A previous meta‐analysis suggests that 52% of cancer survivors report having CRF lasting years post‐diagnosis.^7^ Although CRF is the most common and debilitating symptom experienced by cancer patients^10^, ^11^ it is far from the only one. Fear of cancer recurrence (FCR), defined as “fear, worry or concern relating to the possibility that cancer will come back or progress”^12^ is the most prevalent unmet supportive care need reported by up to 79% of cancer survivors.^13^ Similar to fatigue, FCR, if left unaddressed, can persist several years after diagnosis and is associated with lower quality of life and emotional distress.^13^, ^14^, ^15^ Both CRF and FCR are more prevalent in younger patients and, to a lesser extent, in women.^16^, ^17^, ^18^ Lifestyle factors such as cigarette smoking have also been identified as risk factors, with current smokers being more likely to report CRF and FCR.^19^

Although FCR and CRF are two common concerns experienced by survivors, their relationship to each other is poorly understood. Previous investigations have been limited by the use of cross‐sectional designs and have reported correlations ranging from 0.30 to 0.55.^20^, ^21^, ^22^, ^23^ Pre‐treatment anxiety, a correlate of FCR, predicts increased CRF during and after completion of treatment, suggesting the possibility that FCR experienced early in the cancer‐trajectory contributes to cancer‐related fatigue over time;^4^, ^5^, ^7^ however, models of FCR^24^, ^25^, ^26^ suggest that the presence of physical symptoms (e.g., pain) exacerbates FCR, which has been corroborated by several systematic reviews.^13^, ^14^, ^15^ Thus, CRF could be contributing to FCR. It could also be the case that CRF and FCR mutually influence each other over the cancer trajectory through common psychological processes of catastrophizing and vulnerability to anxiety.^4^, ^5^, ^26^ In support of this hypothesis is a meta‐analysis of mindfulness interventions for cancer patients that found that increased mindfulness skills predicted a reduction in several outcomes, including CRF and FCR.^27^

The goal of the present study is to explore how CRF and FCR influence each other over time. More specifically, this study aims to explore whether CRF reported approximately 1 year after cancer diagnosis is associated with greater FCR over time, whether patients who manifest FCR early in the disease trajectory report greater CRF over time, or whether these concerns mutually influence each other. Because prior research has reported systematic associations between FCR and CRF and age,^7^, ^16^, ^28^ we examined associations between CRF and FCR for younger versus older survivors, controlling for gender,^29^ and smoking status.^30^ Results have the potential to provide empirical evidence about which of these concerns should be addressed as a priority in the management of cancer patients.

## 2 METHODS

### 2.1 Participants and procedure

The data used for this study was gathered by the American Cancer Society's Study on Cancer Survivors‐I (SCS‐I), a national prospective longitudinal study. Surveys were administered by mail or telephone at three time points: Time 1 (T1, *M* = 1.3 years, SD = 0.32), Time 2 (T2: *M* = 2.2 years, SD = 0.34), and Time 3: T3, *M* = 8.8 years, SD = 0.63 post cancer diagnosis. For T1, 6309 individuals completed the survey. Retention rates were 80% for T2 and 70% for T3. Further details on recruitment and overall methodology are available elsewhere.^31^ The Institutional Review Board of Emory University approved these studies for each state, including the Connecticut Department of Public Health Human Investigation Committee. Secondary data analysis approval was obtained from the University of Ottawa Research and Ethics Board (Ottawa, Ontario).

Participants from 25 randomly selected cancer registries across the United States were recruited to partake in this study. To be eligible, individuals had to be over the age of 18, diagnosed with one of the 10 most incident cancers (prostate, female breast, lung, colorectal, urinary bladder, non‐Hodgkin lymphoma, skin melanoma, kidney, ovarian, or uterine cancer), diagnosed with a local, regional or distant surveillance, epidemiology, and end results (SEER) Summary Stage cancer, reside in the particular state at the time of diagnosis, and have been diagnosed during the studies' eligibility period (April 2001–March 2002).^31^ Individuals were ineligible to participate in the study if they were deemed mentally incapable of completing the survey, did not speak English or Spanish, or had a terminal illness.^31^ Additionally, in analyses, we excluded individuals who reported a cancer recurrence, metastasis, or multiple cancers at any of the three waves of data collection (*n* = 184), or who did not have complete data (i.e., at all three waves) on covariates or outcome measures (*n* = 37). These criteria resulted in a final analytic sample of 1395 participants.

### 2.2 Measures

#### 2.2.1 Socio‐demographic and medical variables

Sociodemographic and medical variables assessed at baseline included age, gender, race/ethnicity, family income, and educational level, occupation, and cancer treatments. Cancer type and stage were recorded from SEER registry data and cross‐verified with baseline survey responses. Smoking status was assessed at T3 with the questions “Have you smoked at least 100 cigarettes in your entire life?” and “Do you now smoke cigarettes every day, some days, or not at all?” Participants were categorized as never, former, or current smokers and a dichotomous variable created to indicate current (coded 1) versus nonsmoker (coded 0) status.

#### 2.2.2 Cancer‐related fatigue

CRF was assessed at all three time points using the Profile of Mood States‐Short Form (POMS‐SF) fatigue‐inertia subscale.^32^, ^33^, ^34^ The POMS‐SF has good internal consistency, validity, and reliability^33^, ^34^ (Cronbach alphas >0.89 in samples of cancer patients and controls^35^) and is appropriate for when time or patient ability is limited.^34^ Items are rated on a five‐point response scale ranging from “not at all” to “extremely.” Individuals were required to rate to what extent a certain feeling had been relevant in the past 2 weeks; specifically, “fatigued,” “weary,” “exhausted,” “bushed,” and “worn‐out.” Participants' scores were summed to indicate overall fatigue severity with scores ranging from 0 to 20. In a sample of 437 cancer patients, Baker^32^ reported a mean score for fatigue of 5.63 and established the cut‐off score for clinical classification of an individual as fatigued at ≥6.^33^

#### 2.2.3 Fear of cancer recurrence

FCR was assessed at all three waves using the Cancer Problems in Living Scale's FCR subscale (CIPLS‐FCR).^36^ The CIPLS‐ FCR subscale has good internal consistency (Cronbach alpha = 0.84) and good discriminant validity.^36^ According to a systematic review of FCR measures,^37^ it is an appropriate tool for large cohort studies such as the SCS‐I. Using a four‐point Likert scale (0 = “not at all,” 1 = “somewhat,” 2 = “moderate,” and 3 = “severe”), respondents indicated how much of a problem each of the following was in the last 12 months: “feeling fearful that my illness will return,” “concern about my cancer relapsing,” “fears about the future,” and “preoccupation with being ill.” Item scores were added to determine FCR severity, with a possible range of 0–12. Based on a previous trajectory analysis,^38^ we classified as high FCR individuals with scores >6.

### 2.3 Statistical analysis

#### 2.3.1 Descriptive statistics

Frequencies for categorical variables and means and standard deviations for continuous variables for the three waves were calculated. One‐way ANOVAs followed by pairwise comparisons with Bonferroni correction determined if there were significant differences at each wave (*p* < 0.05) in CRF or FCR as a function of each sociodemographic and medical variables. Correlations between and among CRF and FCR at all waves were calculated.

#### 2.3.2 Multivariate analyses

A multiple group (MG) random intercepts cross‐lagged panel model (RI‐CLPM) assessed prospective associations between CRF and FCR.^39^ Random intercepts CLPMs allow for separation of within‐ and between‐person variance in the presence of individual differences.^40^, ^41^ An MG‐RI‐CLPM consists of four components: (1) a between component, consisting of the random intercepts with the factor loadings constrained to one; (2) a within component, consisting of within‐unit fluctuations with measurement error variances constrained to zero; (3) lagged regressions between the within‐unit components; and (4) covariances for the between and within components. A categorical variable is used as a grouping variable allowing the means, the lagged regression coefficients, the (residual) variances, and the (residual) covariances to differ across the groups. Covariates can be included for all the above components. The grouping variable in the current analysis was self‐reported age, based on a median split at T1 (0 = younger, 1 = older, *n* = 697 and 698, respectively).

Analyses were conducted using Mplus Version 8.2. Default values were selected for the model estimator (maximum likelihood), maximum number of iterations (1000), and convergence criterion (0.500D‐04). All default covariances were set to 0. Fit was assessed with the comparative fit index (CFI), the Tucker‐Lewis index (TLI), the, normative fit index (NFI), and the root‐mean‐square error of approximation (RMSEA). As indication of good model fit, we used values ≥0.95 for CFI, TLI, and NFI and ≤0.06 for RMSEA.^42^, ^43^

## 3 RESULTS

### 3.1 Sample characteristics

In the current sample the mean age was 56.5 (SD = 10.9; median = 55.1), 62.6% were female, 91.8% were Non‐Hispanic White, and 70.3% had at least some college education (Table 1). Approximately three‐quarters (76.6%) of the sample were married at T1, and 25.7% reported annual family incomes of less than $40,000 (Table 1). Cancer sites included breast (32.3%), prostate (20.6%), colorectal (13.3%), uterine (8.0%), skin melanoma (5.9%), NHL (5.4%), kidney (5.2%), ovarian (3.5%), lung (3.4%), and bladder (2.4%). Most participants had localized cancers (70.5%); however, some had regional (23.4%), distant (4.3%) or in situ (1.8%) cancers (Table 1).

**TABLE 1:** Sample characteristics (*N* = 1395).

Sociodemographic and Medical Characteristics	*n* (%)/M(SD)
Age	56.5 (10.9)
Male	522 (37.4)
Female	873 (62.6)
Race/ethnicity	
Non‐Hispanic White	1281 (91.8)
Non‐Hispanic Black	51 (4.1)
Hispanic	31 (2.2)
Non‐Hispanic others	26 (1.9)
Educational attainment	
Eighth grade or less	16 (1.1)
Some high school (grade 9–12)	50 (3.6)
High school diploma or GED	349 (25)
Vocational school or some college	369 (26.5)
College degree	332 (23.8)
Professional or graduate school experience	279 (20)
Marital status	
Married	1069 (76.7)
Living in a marriage‐like relationship	38 (2.7)
Divorced	112 (8.2)
Separated	12 (0.9)
Widowed	67 (4.8)
Single, never married	93 (6.7)
Household Income	
Less than $5000	6 (0.4)
$5000–$9999	17 (1.2)
$10,000–$19,999	73 (5.2)
$20,000–$39,999	264 (18.9)
$40,000–$74,999	478 (34.3)
$75,000 or more	397 (28.5)
Cancer type	
Breast	450 (32.3)
Prostate	287 (20.6)
Colorectal	186 (13.3)
Bladder	34 (2.4)
Uterine	111 (8.0)
Melanoma of skin	83 (5.9)
NHL	76 (5.4)
Kidney	72 (5.2)
Lung	47 (3.4)
Ovarian	49 (3.5)
SEER summary stage	
Localized	983 (70.5)
Regional	327 (23.4)
Distal	60 (4.3)
In Situ	25 (1.8)
Smoking status	
Never	726 (52.0)
Former	556 (39.9)
Current	113 (8.1)
Fear of recurrence (means)	
T1	2.4 (1.9)
T2	2.0 (1.8)
T3	2.0 (2.2)
Cancer‐related fatigue (means)	
T1	5.2 (4.4)
T2	5.2 (4.5)
T3	4.4 (4.5)

*Note*: Fear of recurrence = Cancer Problems in Living Scale's Fear of Cancer Recurrence subscale (CIPLS‐FCR); range = 0–12. Cancer‐related fatigue = Profile of Mood States‐Short Form (POMS‐SF) fatigue‐inertia subscale; range = 0–20. Percent may not add up to 100 because of missing data on some variables.

Participants in our analytic sample were somewhat younger in age (at T1) compared to other respondents (56.5 vs. 60.9 years, respectively), and more likely to be female (62.6% vs. 56.4%), Non‐Hispanic White (91.8% vs. 83.6%), educated (70.3% vs. 54.8% with at least some college), married (76.7% vs. 68.1%), and diagnosed with breast (32.3% vs. 20.4%) and uterine (8.0% vs. 4.5%) cancers; however, they were less likely to be diagnosed with lung (3.4% vs. 12.1%) or ovarian (3.5% vs. 7.0%) cancers. Rates were similar for all other cancers (<3%). Our sample also had more localized cancers compared to other respondents (70.5% vs. 55.8%).

Mean CRF scores at T1, T2, and T3, respectively were 5.2 (SD = 4.4), 5.2 (SD = 4.5), and 4.4 (SD = 4.5). The proportion of the sample who were clinically fatigued was 34% (*n* = 475) at T1, 33% (*n* = 461) at T2, and 29.2% (*n* = 408) at T3. Clinical fatigue at T1 was most prevalent among bladder cancer survivors (50%; *n* = 17), followed by survivors of lung (46.8%, *n* = 22), NHL (42.1%; *n* = 32), breast (37.3%, *n* = 168), uterine (34.2%, *n* = 38), kidney (33.3%, *n* = 24), ovarian (32.7%, *n* = 16) and colorectal (32.3%; *n* = 60) cancers. Clinical fatigue was lowest among prostate cancer survivors (26.5%; *n* = 76).

Mean FCR at waves T1, T2, and T3 were 2.4 (SD = 1.9), 2.0 (SD = 1.8), and 2.0 (*SD* = 2.2), respectively. The proportion of participants with a CPILS‐FCR score >6 at T1, T2, and T3 was 3.9% (*n* = 54), 1.9% (26), and 4.4% (*n* = 62), respectively. High FCR at T1 was most prevalent among NHL survivors (13.2%; *n* = 10), followed by survivors of lung (8.5%; *n* = 4), breast (5.1%, *n* = 23), kidney (4.2%, *n* = 3) and skin melanoma (3.6%; *n* = 3) cancers. Rates of high FCR for all other cancers were <3%.

### 3.2 Bivariate associations of CRF and FCR with sociodemographic and medical variables

#### 3.2.1 Cancer‐related fatigue

At all three time points, CRF was greater among younger (<55.1 years) versus older survivors (Table 2). Males reported greater CRF at T1 compared to females, but at T2 and T3, CRF was higher among female survivors. At all three time points, CRF was higher among those with lower household income versus higher income individuals, and among “separated” survivors versus “married” or “widowed” individuals (Table 2). Survivors who were current smokers had consistently higher levels of CRF compared to never or former smokers.

**TABLE 2:** Bivariate associations between sociodemographic and medical characteristics with cancer‐related fatigue (CRF) and fear of cancer recurrence (FCR).

	Cancer related fatigue (CRF)						Fear of cancer recurrence (FCR)					
	T1		T2		T3		T1		T2		T3	
	*M* (SD)/*r*	*p* ^a^	M (SD)/*r*	*p*	M (SD)/*r*	*p*	*M* (*SD*)/*r*	*p*	*M* (SD)/*r*	*p*	*M* (SD)/*r*	*p*
Age												
<55.1	6.0 (4.8)	0.001	5.8 (4.7)	0.001	5.2 (4.8)	0.001	3.0 (1.9)	0.001	2.4 (1.8)	0.001	2.5 (2.5)	0.001
>55.1	4.4 (3.9)		4.6 (4.1)		3.7 (4.1)		1.9 (1.8)		1.6 (1.7)		1.4 (1.8)	
Gender												
Male	4.7 (4.1)	0.001	4.6 (4.0)	0.001	4.0 (4.3)	0.004	2.0 (1.9)	0.001	1.7 (1.7)	0.003	1.5 (1.9)	0.001
Female	2.7 (1.9)		5.5 (4.7)		4.7 (4.6)		2.7 (1.9)		2.2 (1.8)		2.2 (2.3)	
Race/ethnicity												
Non‐Hispanic White	5.2 (4.4)	0.85	5.2 (4.5)	0.44	4.5 (4.5)	0.95	2.4 (1.9)	0.70	2.0 (1.8)	0.38	2.0 (2.2)	0.02
Non‐Hispanic Black	5.0 (4.5)		4.6 (4.3)		4.2 (4.6)		2.2 (2.1)		2.0 (1.8)		1.8 (2.3)	
Hispanic	4.8 (4.3)		6.0 (4.7)		4.1 (4.5)		2.6 (2.0)		2.6 (2.1)		3.1 (3.8)	
Non‐Hispanic others	4.7 (4.3)		6.0 (4.2)		4.4 (4.5)		2.5 (1.7)		1.9 (1.7)		1.5 (1.9)	
Educational attainment												
Eighth grade or less	5.0 (4.1)	0.24	5.5 (3.8)	0.26	5.2 (4.3)	0.43	1.5 (1.7)	0.47	2.1 (1.7)	0.98	1.3 (1.8)	0.64
Some high school (grade 9–12)	5.5 (4.4)		4.9 (4.4)		4.3 (4.0)		2.2 (2.2)		2.1 (2.0)		1.7 (2.1)	
High school diploma or GED	5.3 (4.5)		5.4 (4.6)		4.6 (5.0)		2.4 (2.0)		2.1 (1.8)		2.0 (2.3)	
Vocational school/some college	5.5 (4.5)		5.3 (4.7)		4.7 (4.7)		2.5 (1.8)		2.1 (1.8)		2.1 (2.3)	
College degree	5.1 (4.3)		5.3 (4.5)		4.4 (4.4)		2.4 (2.0)		2.0 (1.8)		2.0 (2.2)	
Professional or graduate school experience	4.7 (4.1)		4.6 (4.0)		4.0 (3.9)		2.4 (1.9)		2.0 (1.7)		1.9 (2.1)	
Marital status												
Married	5.0 (4.3)	0.001	5.0 (4.3)	0.001	4.2 (4.4)	0.001	3.4 (1.9)	0.001	2.0 (1.8)	0.003	1.9 (2.1)	0.001
Living in a marriage‐like relationship	6.5 (4.9)		6.8 (4.7)		6.5 (5.2)		3.9 (2.1)		2.9 (1.9)		3.4 (2.8)	
Divorced	5.9 (4.5)		5.8 (5.0)		5.2 (4.8)		2.5 (2.2)		2.4 (1.8)		2.5 (2.8)	
Separated	8.0 (6.2)		9.5 (6.4)		7.7 (6.4)		3.8 (2.3)		3.0 (1.8)		3.0 (3.9)	
Widowed	4.3 (4.1)		4.8 (4.2)		3.9 (4.6)		2.1 (1.7)		1.8 (1.7)		1.6 (1.9)	
Single, never married	6.1 (5.0)		5.6 (4.9)		5.3 (4.9)		2.6 (1.8)		2.1 (1.7)		2.0 (2.0)	
Household Income												
<$5000	6.5 (5.9)	0.001	6.3 (5.7)	0.001	4.7 (4.2)	0.001	2.3 (2.7)	0.03	2.8 (2.7)	0.02	1.8 (3.3)	0.01
$5000–$9999	9.7 (5.3)		8.8 (4.9)		7.1 (5.3)		3.2 (2.4)		3.1 (2.3)		3.6 (2.9)	
$10,000–$19,999	5.8 (4.7)		6.4 (5.3)		5.1 (5.1)		2.4 (2.1)		2.3 (1.8)		1.8 (2.5)	
$20,000–$39,999	5.1 (4.5)		5.3 (4.8)		4.9 (5.1)		2.1 (2.0)		1.8 (1.8)		1.8 (2.0)	
$40,000–$74,999	5.6 (4.6)		5.4 (4.6)		4.7 (4.7)		2.5 (1.8)		2.0 (1.7)		2.1 (2.3)	
$75,000 or more	4.5 (3.8)		4.4 (3.9)		3.5 (3.7)		2.6 (1.9)		2.1 (1.8)		1.8 (1.9)	
SEER summary stage												
In situ	6.4 (4.4)	0.38	6.0 (5.0)	0.82	5.0 (4.4)	0.82	2.7 (1.7)	0.001	1.7 (1.5)	0.002	1.8 (2.1)	0.01
Localized	5.1 (4.3)		5.2 (4.5)		4.4 (4.5)		2.3 (1.9)		1.9 (1.8)		1.9 (2.1)	
Regional	5.2 (4.5)		5.2 (4.4)		4.6 (4.6)		2.8 (2.0)		2.3 (1.8)		2.3 (2.5)	
Distant	5.7 (4.7)		5.0 (3.8)		4.7 (4.1)		3.0 (2.0)		2.5 (1.9)		2.4 (2.6)	
Smoking status												
Never	5.2 (4.4)	0.04	5.0 (4.4)	0.001	4.4 (4.5)	0.29	2.4 (1.9)	0.22	2.0 (1.7)	0.001	2.0 (2.1)	0.01
Former	5.0 (4.2)		5.0 (4.3)		4.3 (4.4)		2.4 (1.9)		1.9 (1.8)		1.9 (2.2)	
Current	6.1 (4.9)		6.8 (5.2)		5.1 (5.3)		2.7 (2.1)		2.6 (1.9)		2.5 (2.6)	
Correlations												
Cancer related fatigue												
T1	1.0		0.69***		0.54***							
T2			1.0		0.56***							
T3					1.0							
Fear of cancer recurrence												
T1	0.31***		0.25***		0.24***		1.0		0.70***		0.53***	
T2	0.35***		0.33***		0.26***				1.0		0.55***	
T3	0.27***		0.27***		0.33***						1.0	

^a^ *p* values represent significance of one‐way ANOVAs performed at each timepoint.

*** *p* < 0.001.

#### 3.2.2 Fear of cancer recurrence

At all three timepoints FCR was higher among younger versus older survivors, and females versus males. At all three timepoints FCR was lowest among “widowed” survivors and those with in‐situ cancers. Survivors who were current smokers had consistently higher levels of FCR compared to never smokers but only at T2 and T3.

#### 3.2.3 Interrelationships of CRF and FCR

CRF and FCR were positively and significantly correlated at T1 (*r* = 0.31), T2 (*r* = 0.33) and at T3 (*r* = 0.33), all *p* < 001.

### 3.3 MG‐RI‐CLP model

Fit indices indicated good model fit. The *χ* ^2^ test of model fit was (appropriately) not significant, *χ* ^2^ (18) = 23.33, *p* = 0.18. The root mean square error of approximation (RMSEA) was less than 0.02 (90% C.I. = 0.00–0.04). The CFI, the TLI, and the NFI were all large (CFI = 0.99, TLI = 0.99, NFI = 0.99).

**FIGURE 1:**
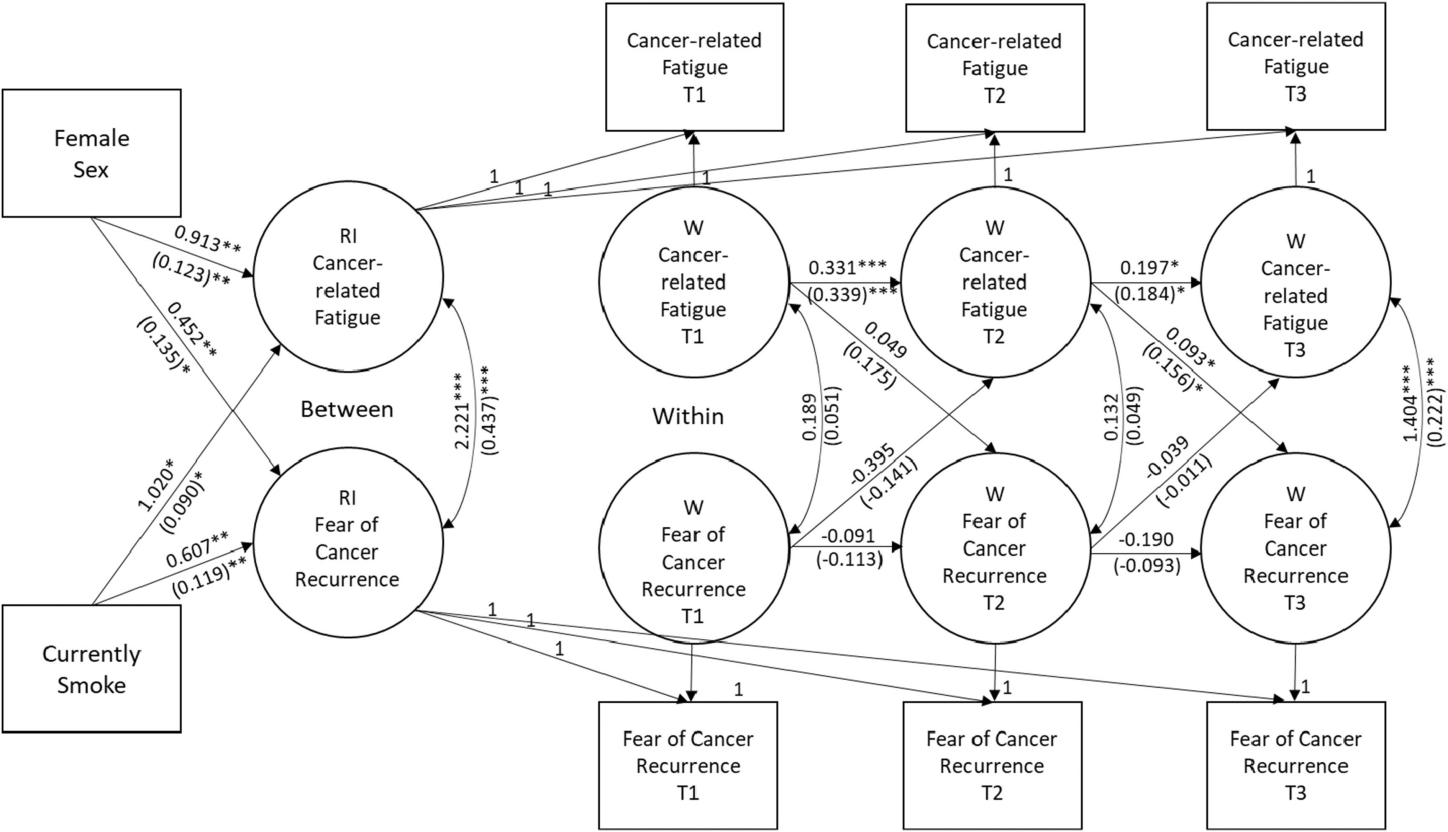
Results of multi‐group random intercepts cross‐lagged panel model for young (<55.1 years) cancer survivors (*n* = 697). Standardized coefficients in parentheses. *p < 0.05, **p < 0.01, ***p < 0.001.

Model results (Figure 1) supported the hypothesis of a lagged effect of CRF on subsequent FCR for participants in the younger age group, but not for older survivors. Among younger participants, higher rates of CRF at T1 showed a trend but did not significantly predict greater FCR at Time 2 (standardized coefficient = 0.18, *p* = 0.09). In addition, higher rates of CRF at Time 2 significantly predicted greater FCR at Time 3 (standardized coefficient = 0.16, *p* = 0.03). The hypothesis of a lagged effect of FCR on subsequent CRF was not supported; none of the coefficients to support that direction of causality were significant for older or younger age groups.

We also tested RI‐CLPMs based on grouping the sample by sex, or by smoking status, controlling for age, sex, or smoking status as appropriate, but none of these models achieved good fit according to fit indices (e.g., RMSEAs >0.06).

## 4 DISCUSSION

CRF and FCR are common and debilitating sequelae of cancer and its treatments; however, more research is needed on how each influences the other over time. Therefore, the main purpose of this study was to explore the relationship between CRF and FCR in cancer patients longitudinally. More specifically, we wanted to investigate whether CRF would lead to increases in FCR, whether FCR would lead patients to become fatigued, or if both CRF and FCR mutually influence each other over time. We also examined the impact of age, sex, and smoking status as possible moderators of the relationship between CFR and FCR. Overall, the results of the present study suggest that CRF may contribute to FCR among younger cancer patients, and that this relationship may become stronger over time. However, the results did not suggest that CRF influences FCR among survivors over age 55 and there were no moderating effects of gender or smoking status (see Figure 1).

The impact of CRF on FCR may be due to lack of awareness about CRF, increased hypervigilance, catastrophization, worse illness perceptions, or decreased self‐efficacy among those who continue to experience CRF over time. One potential explanation for the observed relationship among younger but not older cancer survivors is minimal experience with physical symptoms that increases with age and age‐related conditions among younger survivors. Younger populations are less likely to have experience with age‐related symptoms, such as fatigue and pain, and, therefore, may be more likely to catastrophize their symptoms as being related to their cancer,^44^ or find them to cause more illness intrusiveness,^45^ After success with cancer treatment, patients can become hyperconscious of physical sensations and interpret most physical sensations as being dangerous.^44^ This has been documented with pain and may also apply to CRF. Specifically, hypervigilance to pain increases the perception of pain, making it more disruptive in everyday life. This may not only increase FCR but may also help to maintain it.

Additionally, elevated and persistent CRF could lead to worse illness perceptions. The common‐sense model of illness states that an individual's perception of their illness guides the way they cope with it which impacts the outcomes of the illness (i.e., health outcomes, psychological wellbeing).^46^ Younger survivors may perceive the consequences of CRF as more severe because it interferes with several important life goals, such as starting a family, taking care of young children, or managing work. CRF that does not subside over time may also led to a decreased sense of self‐efficacy around one's health, which contributes to elevated FCR.^47^ Qualitative analyses or think‐aloud protocols comparing younger to older survivors could be a way to further discover the meaning of CRF for survivors and shed light on the age differences we found for the relationship between CRF and FCR.

Regarding the finding that the influence of CRF on FCR was stronger from T2 to T3 as compared to T1 to T2, we can speculate that patients are expecting a certain level of fatigue during or shortly after cancer treatment (i.e., at T1 approximately 1 year after diagnosis). However, they may not receive adequate psychoeducation about CRF from their healthcare providers and may be unaware that this symptom can persist for years following the completion of treatment (i.e., at T2 and T3, approximately 2 and 9 years after diagnosis). This could result in persistent CRF increasing hypervigilance, catastrophization, and worse illness perceptions and decreased self‐efficacy, ultimately impacting FCR among long‐term cancer survivors. A recent study of adult survivors of childhood cancer lends support to this: survivors who had unmet informational needs around CRF also reported greater FCR.^18^ Psychoeducation about what CRF is, its prevalence, persistence, and management along with psychoeducation on the signs and symptoms of recurrence could help patients feel more confident about managing their health and decrease their hypervigilance and catastophization around CRF.

Our finding that CRF subsequently predicted FCR, but not the converse, highlights the importance of early detection and intervention for the management of CRF. With self‐disclosures needed to flag CRF, many patient are left undiagnosed and untreated for this debilitating symptom.^48^ Clinical Practice Guidelines recommend that following a cancer diagnosis, all cancer patients be regularly screened for CRF.^6^, ^49^ However, as of 2020, Jones and colleagues found that CRF guidelines were not regularly implemented in clinical settings, including screening, leaving patients unaware of CFR and how to manage it.^50^ With more efforts on CRF screening and early interventions, it is possible that this could contribute to lower FCR severity. Future studies of CRF should investigate FCR as an outcome of interest.

### 4.1 Limitations and future directions

There were several limitations in this study that will need to be addressed in subsequent research. The sample was collected in 2001–2002 and might not be representative of the general cancer population as a greater proportion of our sample was female (62.6%), non‐Hispanic White (91.8%) and married (76.7%). In addition, the scales used to measure CRF and FCR are brief measures that are limited in their ability to capture multiple dimensions of the variables. The study only includes three time‐points of data collection that were not equally spaced. The first time point took place 1.3 years post cancer diagnosis and thus we could not examine the relationship between CRF and FCR at time of diagnosis and completion of treatment. Our statistical approach of RI‐CLPM also did not allow accounting for differences in time between waves.

Last, fewer participants than anticipated were fatigued and/or had FCR, as per the suggested cut‐off scores on the POMS‐ and CPILS, compared to previous studies, suggesting the possibility of a “healthy survivor” bias, that is patients with lower levels of FCR and/or CRF may have chosen to participate in the study. Additionally, future studies should examine the impact of cancer type on the relationship between CRF and FCR.

## 5 CONCLUSION

The aim of this exploratory study was to investigate how CRF and FCR influence each other through time in a mixed sample of 10 different cancer types. We found a modest effect of CRF on reports of later FCR for younger individuals, but not for survivors over age 55. This study highlights the importance of psychoeducation, early detection, and interventions of CRF in all populations, especially in younger cancer survivors, as persistent CRF may contribute to long‐term FCR.

## AUTHOR CONTRIBUTIONS

**Geneviève Trudel:** Conceptualization (lead); formal analysis (supporting); methodology (supporting); writing – original draft (lead). **Sophie Lebel:** Conceptualization (supporting); formal analysis (supporting); methodology (supporting); project administration (equal); resources (lead); supervision (lead); writing – original draft (supporting); writing – review and editing (equal). **Robert L. Stephens:** Formal analysis (lead); validation (lead); writing – original draft (supporting); writing – review and editing (supporting). **Caroline Séguin Leclair:** Conceptualization (supporting); data curation (supporting); formal analysis (supporting); methodology (supporting). **Corinne R. Leach:** Resources (supporting); writing – review and editing (supporting). **J. Lee Westmaas:** Conceptualization (supporting); data curation (supporting); formal analysis (supporting); investigation (supporting); methodology (supporting); project administration (equal); resources (supporting); writing – original draft (supporting); writing – review and editing (supporting).

## FUNDING INFORMATION

The American Cancer Society (ACS) Studies of Cancer Survivors (SCS) were funded as an intramural program of research conducted by the ACS Intramural Research Department.

## CONFLICT OF INTEREST STATEMENT

J. Lee Westmaas is employed by the ACS, which receives grants from private and corporate foundations, including foundations associated with companies in the health sector, for research outside the submitted work. This author is not funded by or key personnel for any of these grants, and his salary is solely funded through American Cancer Society funds. Geneviève Trudel, Caroline Séguin Leclair, Bob Stephens, Corinne R. Leach, and Sophie Lebel made no disclosures.

## PRECIS FOR USE IN THE TABLE OF CONTENTS

Data from the American Cancer Society's Study of Cancer Survivors‐I (SCS‐I) was used to examine the relationship between cancer‐related fatigue (CRF) and fear of cancer recurrence (FCR) over 3 time‐points. For participants younger than 55 years of age, CRF predicted FCR over time but no lagged effects of FCR on subsequent CRF were observed.

## Data Availability

Data is available upon request from the corresponding author.
